# Benefits of Multiple-Intervention Pulmonary Rehabilitation to Older Adults with High-Risk Multimorbidity after Coronary Artery Bypass Grafting

**DOI:** 10.3390/healthcare8040368

**Published:** 2020-09-27

**Authors:** Jui-Fang Liu, Hsiu-Mei Lee, Jui-O Chen, Tien-Pei Fang, Yu-Mu Chen, Chien-Ming Lo, Shih-Feng Liu, Hui-Ling Lin

**Affiliations:** 1 Department of Respiratory Care, Chang Gung University of Science and Technology, Chiayi 61363, Taiwan; chin9688@yahoo.com.tw (J.-F.L.); pig61210@gmail.com (T.-P.F.); 2 Department of Education, National Kaohsiung Normal University, Kaohsiung 82444, Taiwan; 3 Department of Respiratory Therapy, Chang Gung Memorial Hospital-Kaohsiung Medical Center and Chang Gung University College of Medicine, Kaohsiung 93301, Taiwan; 0krain@cgmh.org.tw (H.-M.L.); juiobest@tajen.edu.tw (J.-O.C.); 4 College of Nursing, Kaohsiung Medical University, Kaohsiung 80759, Taiwan; 5 Department of Respiratory Therapy, Chiayi Chang Gung Memorial Hospital, Chiayi 61363, Taiwan; 6 Division of Pulmonary and Critical Care Medicine, Department of Internal Medicine, Kaohsiung Chang Gung Memorial Hospital and Chang Gung University College of Medicine, Kaohsiung 83301, Taiwan; blackie@cgmh.org.tw (Y.-M.C.); t123207424@cgmh.org.tw (S.-F.L.); 7 Department of Thoracic and Cardiovascular Surgery, Kaohsiung Chang Gung Memorial Hospital, Chang Gung University College of Medicine, Kaohsiung 83301, Taiwan; liuphysico@yahoo.com.tw; 8 Department of Respiratory Therapy, Chang Gung University, Taoyuan 33301, Taiwan

**Keywords:** coronary artery bypass grafting, multiple-intervention pulmonary rehabilitation, multimorbidity, older adult

## Abstract

Objective: Multimorbidity in elderly patients increases complications and retards the recovery of pulmonary function after coronary artery bypass grafting (CABG) surgery. We aimed to evaluate the impact of multiple-intervention pulmonary rehabilitation (PR) on respiratory muscle strength and dyspnea scores after CABG in adult patients aged ≥65 years who had multimorbidity. Methods: A cohort study was retrospectively conducted with 95 adults aged ≥65 years who underwent CABG surgery and completed a multiple-intervention PR program. Results: Patients in the non-multimorbidity (*n* = 56) and multimorbidity groups (*n* = 39) were evaluated on the basis of their muscle strength, degree of dyspnea, and pulmonary function. Postoperative complications were compared after the completion of PR. Between extubation days 1 and 14, the multimorbidity group showed significant improvements in maximal inspiratory pressure (16.91 vs. 24.95 cmH_2_O, *P* < 0.001), Borg Scale score (0.99 vs. 2.3, *P* < 0.001), and the ratio of forced expiratory volume in 1 s to forced vital capacity (FEV_1_/FVC ratio) of 7.02% vs. 13.4% (*P* = 0.01). The incidence rates of pulmonary complications were similar between the two groups. Conclusions: Multi-interventional PR program significantly improved the maximal inspiratory pressure, Borg scale score, and FEV_1_/FVC ratio in the adult patients aged ≥65 years who had multimorbidity after undergoing CABG surgery.

## 1. Introduction

Multimorbidity refers to the coexistence of two or more chronic diseases in one patient, defined as the concurrent presence of two or more chronic disease in one patient, is the primary is the primary risk factor for adverse effects on health-related quality of life among patients who have undergone coronary artery bypass grafting (CABG) [1,2].

Older adults aged ≥65 years who developed multimorbidity after undergoing CABG surgery tend to exhibit lower pulmonary function, and higher rates of complications and mortality than younger patients. McKellar reported a mortality rate of 7.3% at 30 days after CABG surgery in older adults [3]. Northrup reported that 60% of older adult patients had poor prognosis after heart surgery [4]. A large observational study of 35,173 Chinese patients who underwent CABG surgery reported a significant association between age and long-term mortality of >3 years [5]. A 10-year increment in patient age was associated with a higher mortality rate, with 1.77-, 1.97-, and 2-fold increases in 30-day, 180-day, and 3-year mortality rates, respectively [4].

Age-related changes in respiratory physiology increase the complexity of disease management. Sharma and Goodwin reported age-related effects, including muscle atrophy and weakening of the inspiratory muscles, reduced pulmonary function and compliance, increased respiratory rate, and reduced hemoglobin genesis and immunity [6]. The incidence of pulmonary complications after surgery is higher in older adult patients with multimorbidities such as diabetes mellitus, hyperlipidemia, cerebrovascular disease, chronic obstructive pulmonary disease (COPD), liver disease, chronic kidney insufficiency, and obesity [7,8,9]. Postoperative pulmonary complications, including atelectasis, pleural effusion, diaphragm paralysis, pneumonia, bronchoconstriction, hypoxemia, weakened respiratory muscles, excessive sputum production, prolonged use of mechanical ventilation, pulmonary edema, and respiratory failure, may occur after CABG surgery [7,10,11].

Studies have been demonstrated the following benefits of pulmonary rehabilitation for patients with COPD, reducing dyspnea, increasing exercise capacity, and improving quality of life; thus, pulmonary rehabilitation has been widely adopted for patients with deterioration of pulmonary function [12,13,14,15]. Moreover, pulmonary rehabilitation programs improved pulmonary function and reduced pulmonary complications in patients who underwent CABG surgery [15,16,17,18]. A meta-analysis revealed that perioperative pulmonary rehabilitation reduces postoperative atelectasis [17]. As multimorbidity increases pulmonary complications, we hypothesized that multiple-intervention pulmonary rehabilitation programs after CABG surgery in patients with multimorbidity may facilitate the recovery of pulmonary status and reduce pulmonary complications. Therefore, this study determined the effectiveness of multiple-intervention pulmonary rehabilitation in terms of respiratory muscle strength and pulmonary complications in patients with multimorbidity who experienced CABG surgery.

## 2. Materials and Methods

### 2.1. Study Design and Population

In this single-center, retrospective, observational cohort study, we analyzed data extracted from patient medical records at Kaohsiung Chang Gung Memorial Hospital in Kaohsiung, Taiwan, between January 2009 and December 2013. This study was approved by Chang Gung Medical Foundation Institutional Review Board (IRB103-0898C), and the approval included a waiver of the need for informed consent. Consecutive patients who had undergone CABG surgery and completed a routine multiple-intervention pulmonary rehabilitation program during hospitalization were included in the first screening.

**Figure 1:**
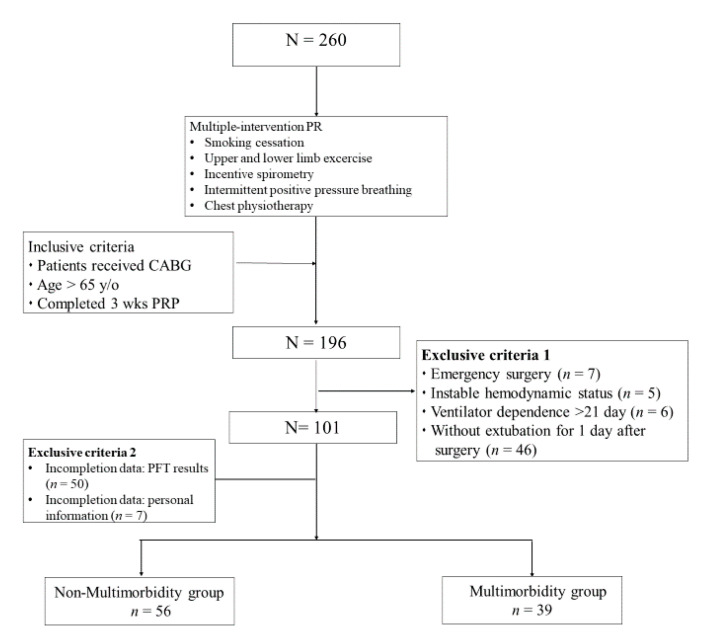
Overview of patient selection. Abbreviations: PRP: pulmonary rehabilitation program; PFT: pulmonary function test.

Patients who had undergone emergency cardiac surgery and those who were hemodynamically unstable were excluded. Generally, patients undergoing CABG surgery are extubated and removed from mechanical ventilation within 24 h; therefore, patients who have received invasive mechanical ventilation for >1 day, indicating complications from the surgery or related to heart function, were excluded. We collected patient data on demographic characteristics, muscle strength, degree of dyspnea, pulmonary function, and postoperative pulmonary complications [19]. Patients with incompletely listed parameters were excluded. Patients were assigned to a multimorbidity group if they had at least two comorbidities, such as COPD, diabetic mellitus, hypertension, chronic kidney disease, or cerebrovascular accident; otherwise, they were assigned to a non-multimorbidity group (Figure 1).

### 2.2. Multiple-Intervention Pulmonary Rehabilitation Program

All patients who undergo thoracic surgery at Kaohsiung Chang Gung Memorial Hospital routinely receive multiple-intervention pulmonary rehabilitation consisting of smoking cessation and breathing control education, including pursed-lip breathing, diaphragmatic breathing, and cough training; upper and lower limb exercises from 30 min per day to patient tolerance, as determined using the modified Borg scale score; incentive spirometry three times per day; intermittent positive pressure breathing for 10–15 min per day; and chest physiotherapy three times per day (manual percussion and mechanical vibration on the chest wall while the patient performed huffing with the vocal cords open, and postural drainage by a respiratory therapist). The interventions commenced 1 week preoperatively and sustained for 2 weeks postoperatively immediately after extubation on day 1.

### 2.3. Data Collection

Demographic characteristics of patients included age, sex, body mass index, smoking index, severity of coronary artery disease, and surgical procedure. Postoperative complications included days of oxygen use from the point of extubation to breathing room air; pulmonary-related complications such as respiratory failure, atelectasis, pneumonia, and barotrauma such as pneumothorax and subcutaneous emphysema; and other complications such as urinary tract infection, wound infection, reparative surgery, and bacteremia.

We recorded patients’ pulmonary status, including respiratory muscle strength, degree of dyspnea, and pulmonary function, on extubation days 1 through 14. The modified Borg scale was used to evaluate self-perceived degree of dyspnea, rated by patients on a scale of 0–10, [20]. The patients rated their degree of dyspnea on a scale of 0–10, where 0 denoted no breathlessness at all and 10 denoted maximum breathlessness. The maximal inspiratory pressure (MIP; cmH_2_O), maximal expiratory pressure (MEP; cmH_2_O), and respiratory rate (breath/min) were obtained from the medical charts. The pulmonary function of the patients was evaluated using spirometry on postoperative days 1 and 14. The parameters measured included forced vital capacity (FVC), forced expiratory volume in 1 s (FEV_1_), FEV_1_/FVC ratio, peak expiratory flow, and forced expiratory flow between 25% and 75% of vital capacity (FEF_25–75%_).

### 2.4. Statistical Analyses

A normality test was performed, and data were presented as mean ± standard deviation (SD) for continuous variables and as number (percentage) for categorical variables. A chi-square test was used to analyze demographic data. A Mann-Whitney U test was conducted to compare the variables on postoperative days 1 and 14. A Wilcoxon signed-rank test was performed to compare changes in the variables on postoperative days 1 and 14 between the multimorbidity and non-multimorbidity groups. Pearson’s correlation coefficient was conducted for relationship between Borg scale score and FEV_1_/FVC. All *P* values were two-tailed with a statistically significant level of less than 0.05. Statistical analyses were performed using the Statistical Package for the Social Sciences version 23.0 (SSPS Inc., Chicago, IL, USA).

## 3. Results

During the study period, 260 patients aged ≥65 years underwent CABG surgery and completed a 3-week multiple-intervention pulmonary rehabilitation program (Figure 1). Among these patients, 64 were excluded because they met the first exclusion criterion, including seven patients with emergency surgery, five patients with unstable hemodynamic status, six patients with ventilator use over 21 days, and 46 patients who could not be extubated at the first of surgery. Furthermore, 101 patients were excluded because of incomplete information, including 51 patients with incomplete PFT reports and 50 patients with missing data of partial patient characteristics. Finally, a total of 95 patients were included in this study, 56 in the non-multimorbidity group and 39 in the multimorbidity group.

### 3.1. Patients’ Characteristics and Complications

The baseline patient characteristics are shown in Table 1. The age difference between the two groups was not significant (*P* = 0.056). Approximately 50% of the patients in both groups were overweight (body mass index, >25 kg/m^2^), and most patients had three-vessel coronary arterial disease and had undergone isolated CABG surgery.

Table 2 presents the oxygen support status and complications after CABG surgery in the two groups. Most patients were free from oxygen support in <7 days. The most common complications in the multimorbidity and non-multimorbidity groups were atelectasis (26.8% vs. 43.6%) and urinary tract infections (26.8% vs. 38.5%), respectively, and the incidence rates did not differ significantly between the two groups.

### 3.2. Improvement of Respiratory Parameters

**Figure 2:**
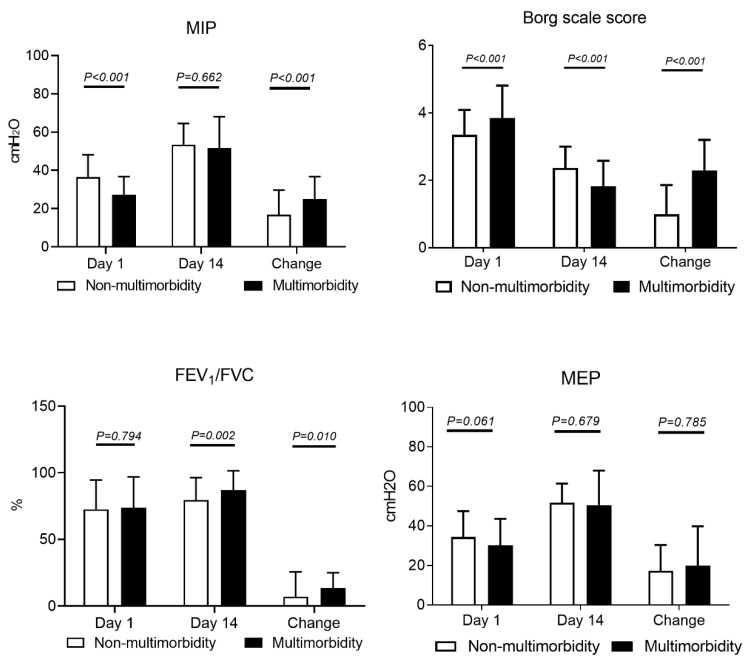
Comparison of respiratory muscle strength and dyspnea degree. MIP: maximum inspiratory pressure; MEP: maximum expiratory pressure

Comparisons of inspiratory muscle strength and dyspnea assessment between the two groups are presented in Figure 2. Patients in the multimorbidity groups exhibited significantly lower MIP between extubation days 1 and 14 (−26.69 vs. −36.52 cmH_2_O, FEV_1_ < 0.001). The improvement in MIP between days 1 and 14 was significantly greater in the multimorbidity group than in the non-multimorbidity group (24.95 vs. 16.91 cmH_2_O, *P* < 0.001).

The Borg scale scores in the multimorbidity group was significantly higher on day 1 (3.85 vs. 3.34, *P* < 0.001), but the change in the score between extubation days 1 and 14 was significantly lower in the multimorbidity group than in the non-multimorbidity group (2.3 vs. 0.99, *P* < 0.001). In addition, a significant change in FEV_1_/FVC ratio between extubation days 1 and 14 was noticed in the multimorbidity group (13.4% vs. 7.02%, *P* = 0.010).

We further evaluated the impact of age and gender to subgroups on MIP, Borg scale score, and FEV_1_/FVC (Table 3). Results showed significant improvement of Borg scale score (*P* = 0.006) in patients aged >75 in multimorbility group while no significant different in non-multimorbility group. Similarly, the greatest improvement on Borg scale score was found in the female in the multimorbility group. The correlation coefficient between Borg scale score and FEV1/FVC was −0.074 (*P* = 0.486).

## 4. Discussion

Our finding suggests that the multiple-intervention pulmonary rehabilitation program was associated with considerable improvements in MIP, Borg scale score, and FEV_1_/FVC ratio in the patients who sustained CABG surgery, particularly those with multimorbidity. The incidence rates of postoperative pulmonary complications were similar between the multimorbidity and non-multimorbidity groups.

Comorbidity has been reported to be the primary risk factor of death following CABG surgery [1]. In addition, patients aged ≥65 years are at a higher risk of complications, reduced pulmonary function, and death after CABG surgery than those aged <65 years [21]. Respiratory muscle strength and gas exchange capacity declines by approximately 15–20% immediately after CABG surgery, resulting in dyspnea sensations. Savci and colleagues reported that inspiratory muscle training led to the rapid recovery of inspiratory muscle strength and functional capacity after CABG surgery [22]. Hulzebos and colleagues confirmed that inspiratory muscle training before CABG surgery reduced the duration of mechanical ventilator use [18]. In our study, the patients who underwent the multiple-intervention pulmonary rehabilitation program exhibited significant improvements in muscle strength and dyspnea score between extubation days 1 and 14. The MIP and Borg scale scores were greatly lower in the multimorbidity group than in the non-multimorbidity group on day 1. Patients in the multimorbidity group exhibited greater improvement than those in the non-multimorbidity group after the pulmonary rehabilitation program on extubation day 14. The breathing exercises, pursed-lip breathing, diaphragmatic breathing, and cough training in the pulmonary rehabilitation program may have contributed to the greater improvements in the degree of dyspnea and MIP. Most pulmonary rehabilitation studies have focused on the benefits of single-intervention designs such as inspiratory muscle training, intermittent positive pressure breathing, or incentive spirometry; however, the multiple interventions in this study achieved improvements in frail patients aged ≥65 years who had multimobilidty [13,22,23].

Reduced pulmonary function has been reported in terms of FEV_1_ and increased residual volume in elderly patients after surgery, resulting in hypoventilation, dyspnea, and impaired secretion clearance; these symptoms worsen with multimorbidity [24,25]. Similarly, our results showed significantly lower MIP and Borg scale scores in patients with multimorbidity on extubation day 1 than in those without multimorbidity. However, the improvements in pulmonary function in terms of FEV_1_/FVC and vital capacity were remarkably greater in patients with than in those without multimorbidity. After CABG surgery, patients might have naturally recovered between extubation days 1 and 14; thus, patients in the multimorbidity group, who had poorer baseline characteristics than those in the non-multimorbidity group, might have exhibited lower values on day 14.

Pulmonary rehabilitation programs provided to patients after cardiac and major abdominal surgeries have been shown to reduce pulmonary ventilation impairment and the incidence of pulmonary complications [26,27]. In a meta-analysis, Katsura reported that inspiratory muscle training reduced the incidence of atelectasis and pneumonia following cardiac and major abdominal surgeries [28]. Additionally, the incidence of pulmonary complications in pneumonia is 13%. In our study, the initiation of multiple interventions in the pulmonary rehabilitation program was intended to reduce the incidence of pulmonary complications after CABG surgery. This multiple-intervention pulmonary rehabilitation program with aggressive lung expansion therapy, airway secretion clearance, and mobilization of the extremities may lower pulmonary complications in both patients with and without multimorbidity. Our results revealed that the incidence of pneumonia was approximately 9.5%, and that of respiratory failure was 1%. Multiple-intervention pulmonary rehabilitation programs are encouraged for patients undergoing CABG surgery.

Our study has some limitations; the external validity of this single-center study should be confirmed through multicenter studies. The sample size of this study is considered small. We calculated Cohen’s d test effect size of the outcomes, resulting in 0.66 for MIP, 1.18 for Borg scale score, 0.411 for FEV_1_/FVC, and 0.14 for FVC [29,30]. The effect size of the Borg dyspnea score is considered large, in which could be interpreted as noticed improvement of dyspnea score from the pulmonary rehabilitation program regardless the sample size. Chronic respiratory disease may greatly impact the lung function recovery during the pulmonary rehabilitation program. Although patients’ characteristics showed no significant different on their previous diagnosis with COPD, a pulmonary function test should be confirmed immediately before the CABG surgery. Additional large-scale clinical prospective studies with long follow-up periods are warranted to validate our findings. To prove the correlation between respiratory functional parameters and quality of life after CABG surgery, the use of specific questionnaires such as Short Form 36 is recommended in the future.

## 5. Conclusions

Multiple-intervention pulmonary rehabilitation was associated with significant improvements in MIP, Borg scale score, and FEV_1_/FVC ratio in elderly patients with multimorbidity who underwent CABG surgery in this study. To facilitate postoperative respiratory recovery, the multiple-intervention pulmonary rehabilitation program is recommended for patients with multimorbidity who undergo CABG surgery.

## Figures and Tables

**Table 1:** Patient characteristics.

Characteristics	Non-Multimorbidity Group (*n* = 56)	Multimorbidity Group (*n* = 39)	*P*-Value
Age (years)	72.20 ± 7.96	69.18 ± 3.62	0.056 ^a^
65–74	40 (71.4%)	34 (87.2%)	
≥75	16 (28.6%)	5 (12.8%)	
Sex			
Male	40 (71.4%)	31 (79.5%)	0.260 ^a^
Female	16 (28.6%)	8 (20.5%)	
Body mass index (kg/m^2^)			
≤24	23 (41.1%)	19 (48.7%)	0.406 ^a^
≥25	33 (58.9%)	20 (51.3%)	
Smoking index (pack-year)	10.00 ± 20.53	14.36 ± 33.54	0.976 ^b^
Coronary artery disease			0.286 ^a^
≤Two-vessel CAD	22 (39.28%)	18 (47.4%)	
≥Three-vessel CAD	34 (60.7%)	21 (52.6%)	
Surgery type			0.572 ^a^
Isolated CABG	42 (75.0%)	26 (68.4%)	
CABG + aortic valve	5 (8.9%)	4 (10.5%)	
CABG + mitral valve	6 (10.7%)	3 (7.9%)	
CABG + ventricular Septal repair	3 (5.4%)	5 (13.2%)	
ASA surgical risk classification			0.007 ^a^
≤3	51 (91.1%)	27 (69.2%)	
≥4	5 (8.9%)	12 (30.8%)	
DM			<0.001 ^a^
No	56 (100.0%)	16 (41.0%)	
Yes	0 (0%)	23 (59.0%)	
Hypertension			0.002 ^a^
No	35 (62.5%)	12 (30.8%)	
Yes	21 (37.5%)	27 (69.2%)	
Chronic kidney disease			<0.001 ^a^
No	56 (100.0%)	27 (69.2%)	
Yes	0 (0%)	12 (30.8%)	
Cerebral vascular accident			<0.001 ^a^
No	56 (100.0%)	31 (79.5%)	
Yes	0 (0%)	8 (20.5%)	
COPD			0.378 ^a^
No	22 (46.4%)	16 (41.0%)	
Yes	30 (53.6%)	23 (59.0%)	

^a^ Chi-square test; ^b^ Mann–Whitney U-test. ASA: American Society of Anesthesiologists; COPD: chronic obstructive pulmonary disease; DM: diabetes mellitus; CAD: coronary artery disease; CABG: coronary artery bypass grafting.

**Table 2:** Postoperative outcomes in patients with and without multimorbidity.

Outcomes	Non-Multimorbidity Group (*n* = 56)	Multimorbidity Group (*n* = 39)	*P*-Value
ICU stay (days)	3.13 ± 2.48	4.98 ± 1.9	0.003
Hospital stay (days)	19.73 ± 6.10	27.18 ± 11.2	<0.001
Oxygen use (days)			0.080
<7 days	48 (85.7%)	28 (71.8%)	
>7 days	8 (14.3%)	11 (28.2%)	
Pulmonary complications			
Respiratory failure	0 (0%)	2 (5.1%)	0.166
Atelectasis	15 (26.8%)	17 (43.6%)	0.069
Pneumonia	5 (8.9%)	4 (10.3%)	0.548
Subcutaneous emphysema	1 (1.8%)	4 (10.3%)	0.089
Pneumothorax	2 (3.6%)	1 (2.6%)	0.665
Other complications			
Urinary tract infection	15 (26.8%)	15 (38.5%)	0.164
Wound infection	3 (5.4%)	4 (10.3%)	0.305
Re-operation	3 (5.4%)	2 (5.1%)	0.332
Bacteremia	1 (1.8%)	0 (0%)	0.589

**Table 3:** Comparisons respiratory muscles change in subgroups.

Parameters	Non-Multimorbidity Group (*n* = 56)			Multimorbidity Group (*n* = 39)		
Age (year)	65–74	>75	*P*-Value	65–74	>75	*P*-Value
MIP	15.9 ± 13.6	17.8 ± 12.0	0.569	25.5 ± 9.3	24.7 ± 13.9	0.915
Borg	−0.8 ± 1.0	−1.2 ± 0.65	0.151	−1.6 ± 0.8	−2.4 ± 0.8	0.006
FEV_1_/FVC (%)	8.2 ± 19.9	5.9 ± 17.7	0.666	12.5 ± 12.2	14.2 ± 11.3	0.665
Gender	Male	Female		Male	Female	
MIP	16.2 ± 12.6	18.6 ± 13.4	0.500	24.4 ± 11.3	27.2 ± 12.8	0.564
Borg	−0.75 ± 0.9	−1.5 ± 0.5	0.001	−1.8 ± 0.8	−3.0 ± 0	0.001
FEV_1_/FVC (%)	14.0 ± 16.3	8.7 ± 13.9	0.001	12.3 ± 12.2	17.5 ± 7.9	0.267

MIP: maximum inspiratory pressure; FEV_1_/FVC: forced expiratory volume in 1 s to forced vital capacity.
